# Lyophilized amniotic membrane patch (LAMPatch) as a replacement of tamponades in the treatment of primary rhegmatogenous retinal detachment

**DOI:** 10.1186/s40942-020-00264-7

**Published:** 2020-11-19

**Authors:** Mario Saravia, Luis Zeman, Alejandro Berra

**Affiliations:** 1 Buenos Aires Mácula-Clinical Research, Buenos Aires, Argentina; 2 Department of Ophthalmology, Hospital de Clínicas, Universidad de Buenos, 2351 Córdoba Ave, Buenos Aires, Argentina; 3 Ocular Investigation Laboratory, Department of Pathology, School of Medicine, University of Buenos Aires, Buenos Aires, Argentina

**Keywords:** Amniotic membrane dressings, Retina, Retinal detachment, Vitreous body, Vitreoretinal surgery, Vitrectomy

## Abstract

**Background:**

The basis of retinal detachment repair is sealing the retinal breaks. In order to seal the retinal breaks, chorioretinal adhesion around these lesions has to be achieved. Laser retinopexy is not immediate thus necessitates the use of a temporal endotamponade to maintain both tissues in apposition. We propose the use of a patch of lyophilized human amniotic membrane (LAMPatch) in order to occlude the retinal tear effectively until the chorioretinal adhesion is settled, overcoming the risks and limitations of the current tamponades.

**Methods:**

23-gauge vitrectomy was performed on eyes with primary retinal detachment with single retinal breaks of less than one-hour extension. A LAMPatch was deployed over the retinal breaks after retina was repositioned with perfluorocarbon. Neither gas nor silicon oil were injected.

**Results:**

Six eyes of six patients with total or partial retinal detachment were included. Retinas remained reattached in all cases until the end on follow-up (3, 5 months). Best-corrected visual acuity at 1-week postop was between 20/30 and 20/100. Neither elevations of intraocular pressure, cataracts nor signs of inflammation were registered during follow-up. No second surgeries were needed.

**Conclusion:**

This technique has proven to be safe and effective in this small case series. No intraocular pressure rise, inflammation or cataracts were registered until last follow-up visit.

## Background

The basis of retinal detachment (RD) repair was established by Jules Gonin 100 years ago and it is still up to date: sealing the retinal break [1]. Vitrectomy has become the prevalent surgical technique, with a reattachment success rate of approximately 85–95%, regardless of the final visual acuity or side effects [2].

In order to seal the retinal breaks, chorioretinal adhesion around these lesions has to be achieved. Laser retinopexy is the preferred technique, nevertheless its effect is not immediate [3], thus necessitates the use of a temporal endotamponade to maintain both tissues in apposition. Long-acting gases and silicone oil are widely used to accomplish this purpose; nonetheless, they are not exempt from side effects, namely, visual acuity impairment during tamponade, cataracts [4] and glaucoma [5] among others. Furthermore, due to their physical properties, they are prone to fail in the context of inferior retinal breaks, and hence, require prolonged anti-natural head positions, which are difficult to achieve by patients.

Human amniotic membrane (HAM) has been reported to be safe for intraocular use; moreover, as Rizzo et al. described, it appears to promote retinal healing [6–8]. Lyophilization of HAM maintains its histological structure [9] and, at the same time, confers beneficial features regarding its storage, biosafety and surgical handling.

We propose the use of a patch of lyophilized HAM (LAMPatch) over the retinal tear as a localized temporary tamponading agent in replacement of currently used tamponades, in order to occlude the retinal tear effectively until the chorioretinal adhesion is settled, overcoming the risks and limitations of the current tamponades.

## Materials and methods

This is a consecutive, single-center case series of six eyes of six patients with primary RD treated by a single surgeon with pars plana 23-gauge vitrectomy with the Constellation System (Alcon Laboratories, Inc., Fort Worth, TX, USA). Inclusion criteria were: eyes with primary RD, single retinal breaks of less than one hour extension and minimal to no signs of vitreoretinal proliferation. Patients with previous retinal surgeries were excluded. Three cases had superior breaks, and three cases had inferior breaks. Surgical repair took place between November 2019 and June 2020. Written informed consent for participation was obtained from all patients. The study was in compliance with the Declaration of Helsinki. An independent ethical committee approved this study in accordance with the Helsinki declaration.

Preoperatively, an ophthalmic history and a complete ophthalmic examination including refraction with assessment of best-corrected visual acuity (BCVA) Goldmann applanation tonometry and a fundus dilated ophthalmic examination was performed.

### HAM lyophilization process

HAMs were obtained from Amnios, Inc, (Buenos Aires, Argentina) where HAM is harvested from human placentas following standardized procedures. A sample of HAM is previously collected to perform Protein Chain Reaction (PCR) for HIV, Hepatitis B, Hepatitis C, Brucellosis, Toxoplasmosis, CMV, Syphilis and Chagas before dispensation. Once the procedure of cleansing and dissecting of the HAM is finished, fragments of different sizes are loaded into sterile Petri dishes, wich are placed in a freezer at −80 ℃ for 2 h. Then, the freezed plates are stored in the freeze-dryer shelves under a determined vacuum (less than 15 Pascals). The final products are exposed to gamma radiation (15–20kgray) for sterilization. (Process validated by the Argentinian National Atomic Energy Commission).

### Surgical procedure

**Fig. 1:**
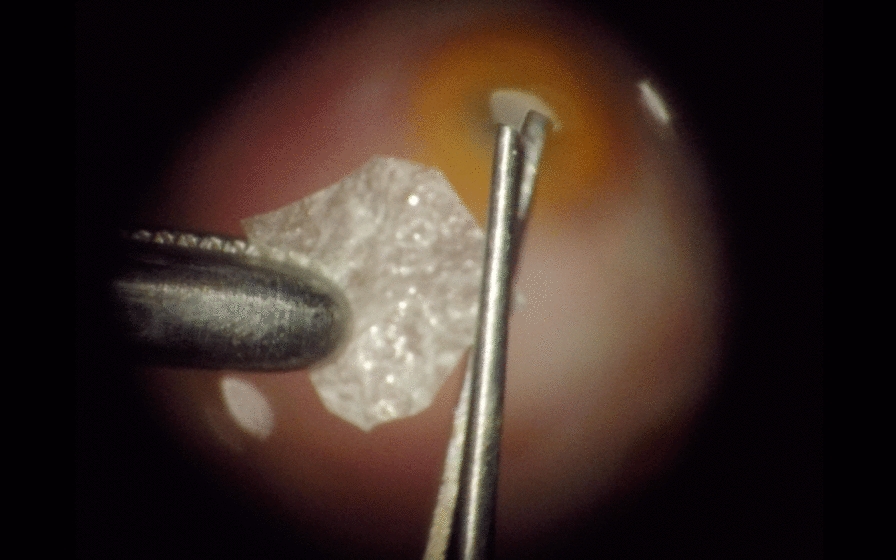
LAMPatch trimming under optical microscope before been introduced inside the eye

**Fig. 2:**
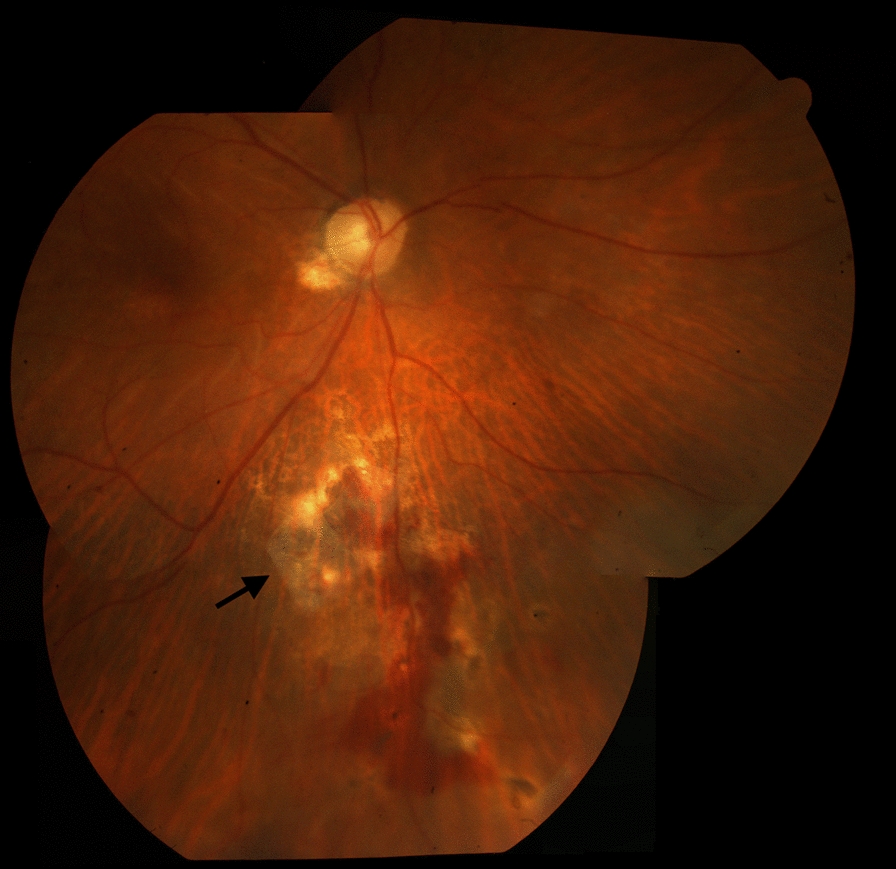
LAMPatch (Black arrow) adhered to the retinal surface covering the retinal break

M.S. performed all surgical procedures with the Alcon Constellation system (Alcon Laboratories, Inc, Fort Worth, TX) as follows: A retrobulbar block was performed followed by placement of three 23-gauge trocars. A complete vitrectomy including a meticulous peripheral vitreous shave was performed. Retina was repositioned with either liquid perfluorocarbon or air ensuring that the borders of the lesions were completely attached and no subretinal fluid was left. Laser photocoagulation was applied around the retinal breaks. Perflurocarbon–liquid–air exchange was performed. Once the eye was full of air, tailored pieces of lyophilized HAM between 3 to 6 mm in diameter (Fig. 1) were introduced using a 25-gauge forceps through a 23-gauge valved trocar (mismatched caliber was intentional for better tissue handling) and deployed over the retinal breaks. As a result of this procedure, a LAMPatch was firmly placed against the retina, covering the break. (Fig. 2) Neither gas nor silicon oil were injected. The trocars were then removed, and the sclerotomies were sutured if needed. Subconjunctival injection of cefazolin was administered.

### Postoperative follow-up

Immediately after surgery, no head-positioning instructions were recommended. Patients were placed on prednisolone acetate 1% and moxifloxacin 0.5% four times daily. All patients were examined postoperatively at the first postoperative day and then at 1-week; the following visits were scheduled on a monthly basis. Mean follow-up was 3.5 months (2–7 months). The postoperative evaluations included measurement of the BCVA and intraocular pressure (IOP), biomicroscopy and dilated fundus examination.

## Results

Six eyes of six patients with total or partial RD were included (Table 1).

**Table 1:** Patient demographics

Patient	Age	Sex	Type of RD	Tear location	Observations	POW1* BCVA	POM1** IOP
1	59	M	Total, macula-off	Superior	None	20/80	18
2	47	F	Partial, macula-off	Superior	High myope	20/60	14
3	51	F	Partial, macula-on	Inferior	None	20/30	12
4	60	F	Partial, macula-off	Inferior	None	20/50	12
5	67	M	Total, macula-off	Superior	None	20/100	14
6	52	F	Partial, macula-off	Inferior	None	20/40	14

This table includes patient demographics, measurements of visual acuity and intraocular pressure and characteristics of retinal detachments

^*^POW1 Postoperative Week 1

^**^POM1 Postoperative Month 1

**Fig. 3:**
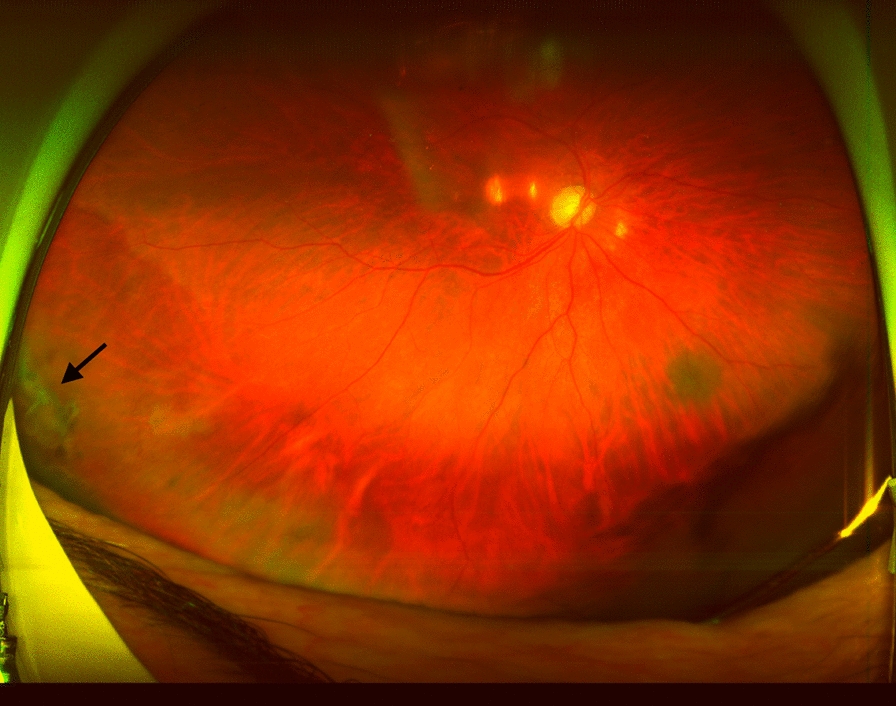
Wide-field retinography of patient 6 (see Table 1) at 5-months. A Black arrow shows LAMPatch in place, partially detached from its borders

Retinas remained reattached in all cases from 1-day to the end of follow-up. All LAMPatches stayed completely adhered to the retinal surface covering the break at the 1-week postoperative visit. In one case the LAMPatch was not in place in the vitreous cavity after a comprehensive fundoscopy. Patient did no referred symptoms at the 1-month postoperative visit (Patient 2). Neither was found in the vitreous cavity after a comprehensive fundoscopy. Patient did not refer any symptoms. In another case (Patient 6) the LAMPatch was found partially detached from its borders at the 5-month postoperative visit (Fig. 3). The other patches remained attached until the end of follow-up. RD did not occur at any time. BCVA at 1-week postop was between 20/30 and 20/100. Neither elevation of IOP, cataracts nor signs of inflammation were registered during follow-up. No second surgeries were needed.

## Discussion

Vitrectomy has become the preferred surgical technique for addressing RD. Technological advances regarding vitrectomy and visualization systems have improved overwhelmingly in the last decades, allowing the procedure to be faster, safer and more comfortable for patients. Notwithstanding, vitrectomy for RD repair still has unwanted side effects due to the use of tamponades. Intraocular silicon oil and/or expansive gases may produce, even when they are no longer inside the eye, visual impairment, cataracts and glaucoma [4, 5]. As the efficacy of the current RD surgery technique (measured by the binary endpoint of “attached/not attached”) is well past 90%, retinal surgeons undertake the described side effects as part of the technique.

We believe that there is room for a different strategy to achieve the same results: to occlude the break and keep retina in position until retinopexy is consolidated, without the side effects caused by current endotamponades. Other papers have been published adressing this issue using a fibrin glue plug, reporting similar benefits [10, 11]. We believe that lyophilized HAM could have additional advantages compared to fibrin glue, given its proven antifibrotic activity [12], a potential protective effect against proliferative vitreoretinopathy, not yet demonstrated.

We propose the use of a thin layer of lyophilized amniotic membrane as a patch (LAMPatch) to temporary block the retinal breaks until a firm laser retinopexy is achieved, avoiding the use of endotamponades like gas or silicon oil. It is critical to understand that this technique does not replace laser retinopexy, conversely, it buys time until strong retinopexy is attained, generally 24 hs postop [3].

HAM has already been used in retinal surgery with good tolerance [8]; nevertheless, as a vital membrane, it has some disadvantages. Firstly, it has to be handled under strict cold chain compliance, resulting in limited viability. Secondly, since fresh HAM contains viable cells, they may also be a way to spread disease vectors. However, after being lyophilized and sterilized by gamma radiation, HAM has no special storage requirements regarding temperature and humidity, negligible risk of biohazard and an enhanced durability.

## Conclusion

In the present series of six cases, LAMPatch technique showed efficacy both in superior and inferior breaks. This technique has proven to be safe and effective in this small case series. An advantage of avoiding the use of endotamponades like gas or silicon oil was that the visual axis was not impaired and visual recovery started earlier. No IOP rise, inflammation or cataracts were registered until last follow-up visit. The limitations of this case series were the short follow-up and the small number of cases.

We propose to consider the use of the LAMPatch technique as an alternative approach for the treament of selected cases of RD.

## Data Availability

All data generated or analyzed during this study are included in this manuscript.

## Abbreviations

RD: Retinal detachment
HAM: Human amniotic membrane
LAMPatch: Lyophilized human amniotic membrane patch
BCVA: Best-corrected visual acuity
PCR: Protein Chain Reaction
IOP: Intraocular pressure
